# An integrated platform for *Brucella* with knowledge graph technology: From genomic analysis to epidemiological projection

**DOI:** 10.3389/fgene.2022.981633

**Published:** 2022-09-14

**Authors:** Fubo Ma, Ming Xiao, Lin Zhu, Wen Jiang, Jizhe Jiang, Peng-Fei Zhang, Kang Li, Min Yue, Le Zhang

**Affiliations:** ^1^ West China Biomedical Big Data Center, West China Hospital, Sichuan University, Chengdu, China; ^2^ College of Computer Science, Sichuan University, Chengdu, China; ^3^ China Animal Health and Epidemiology Center, Qingdao, Shandong, China; ^4^ Department of Medical Oncology, Cancer Center, West China Hospital, Sichuan University, Chengdu, China; ^5^ Med-X Center for Informatics, Sichuan University, Chengdu, China; ^6^ Shanghai Artificial Intelligence Laboratory, Shanghai, China; ^7^ Hainan Institute of Zhejiang University, Sanya, China; ^8^ Key Laboratory of Systems Biology, Hangzhou Institute for Advanced Study, University of Chinese Academy of Sciences, Chinese Academy of Sciences, Hangzhou, China; ^9^ Key Laboratory of Systems Health Science of Zhejiang Province, Hangzhou Institute for Advanced Study, University of Chinese Academy of Sciences, Hangzhou, China

**Keywords:** *Brucella*, genomic analysis, epidemiology, databases, visualization

## Abstract

**Motivation:** *Brucella*, the causative agent of brucellosis, is a global zoonotic pathogen that threatens both veterinary and human health. The main sources of brucellosis are farm animals. Importantly, the bacteria can be used for biological warfare purposes, requiring source tracking and routine surveillance in an integrated manner. Additionally, brucellosis is classified among group B infectious diseases in China and has been reported in 31 Chinese provinces to varying degrees in urban areas. From a national biosecurity perspective, research on brucellosis surveillance has garnered considerable attention and requires an integrated platform to provide researchers with easy access to genomic analysis and provide policymakers with an improved understanding of both reported patients and detected cases for the purpose of precision public health interventions.

**Results:** For the first time in China, we have developed a comprehensive information platform for *Brucella* based on dynamic visualization of the incidence (reported patients) and prevalence (detected cases) of brucellosis in mainland China. Especially, our study establishes a knowledge graph for the literature sources of *Brucella* data so that it can be expanded, queried, and analyzed. When similar “epidemiological comprehensive platforms” are established in the distant future, we can use knowledge graph to share its information. Additionally, we propose a software package for genomic sequence analysis. This platform provides a specialized, dynamic, and visual point-and-click interface for studying brucellosis in mainland China and improving the exploration of *Brucella* in the fields of bioinformatics and disease prevention for both human and veterinary medicine.

## 1 Introduction

Brucellosis is one of the most prevalent infectious diseases worldwide; it is common in low- and middle-income countries, including China, and infects both humans and animals. It is caused by *Brucella*, a Gram-negative bacterium that does not form spores and is generally non-motile. Brucellosis is often linked to animal reservoirs and has significant zoonotic potential. *Brucella* spp. Could infect humans through animals via different routes of contact (Assenga et al., 2015), including mucous membranes, the gastrointestinal tract, the respiratory tract, abraded skin, or the consumption of foods of animal origin (Zhou et al., 2020).

Detection of brucellosis is conducted by various methods, including cultural isolation, serodiagnosis, and molecular diagnosis (Yagupsky et al., 2019). Besides, researchers have used bioinformatics approaches to explore brucellosis based on the molecular biological properties of *Brucella*, such as by computationally analyzing the surface properties of *Brucella* outer membrane protein for vaccine design (Chen et al., 2021) or the correlation of the virulence with structural features of *Brucella* subtypes (Paci et al., 2020). At present, the development of sequencing technologies has obvious advantages in providing digital data in a cost-effective manner (Mardis, 2017) as well as in delivering high-resolution sequencing results, which is a portable precondition for comparative purposes. Next-generation sequencing followed by bioinformatics analysis (Zhang et al., 2019c; Fan et al., 2019; Liu G.-D. et al., 2020; Badai et al., 2020; Liu G. et al., 2020; Zhao et al., 2020; Wolkenhauer, 2021; You et al., 2022) has become the gold standard for epidemiological analysis of pathogenic microorganisms, including zoonotic bacteria.

The established approach for streamline analysis is well recognized. First, bacterial genomic DNA is extracted and subjected to sequencing according to the guidelines of the commercial platform; then, the obtained results are analyzed using bioinformatics software (Zhang et al., 2019a; Zhang et al., 2019d; Lei et al., 2020; Wu et al., 2020; You et al., 2020; Zhang et al., 2021a; Gao et al., 2021; Lei Zhang et al., 2021) or suitable platform databases (Xiao et al., 2020; Zhang et al., 2021b; Xiao et al., 2021) for the assembly and annotation of genomes, determination of SNPs, identification of antimicrobial resistance genes (Lv et al., 2021) and virulence-associated factors, building phylogenetic trees, etc. (Seemann, 2014; Johansen et al., 2018; Yasuhiro et al., 2018; Ledwaba et al., 2021; Liu et al., 2021). However, massive sequencing has led to the accumulation of genomes in databases without optimal exploration, which requires the development of suitable analysis software and integrated platforms to better understand the dynamic epidemiology of zoonotic bacteria.

Although analytic software for either genomic or epidemiological purposes is available, such as the software for genome alignment (Marçais et al., 2018), phylogenetic tree construction (Minh et al., 2020) and gene similarity comparison (Krzywinski et al., 2009), the analytic approach always takes the form of a static data presentation, lacking a dynamic data presentation and an interactive component. By integrating five commonly used genome analysis applications (Shovill, Prokka, Snippy, ABRicate and FastTree) for the study of *Brucella*, we can provide the user with a friendly and interactive graphical interface. Furthermore, national-scale, spatiotemporal surveillance databases are generally lacking in most countries throughout the world.

To address these existing limitations, we developed a comprehensive information platform for *Brucella*, which has two main innovations.

**FIGURE 1:**
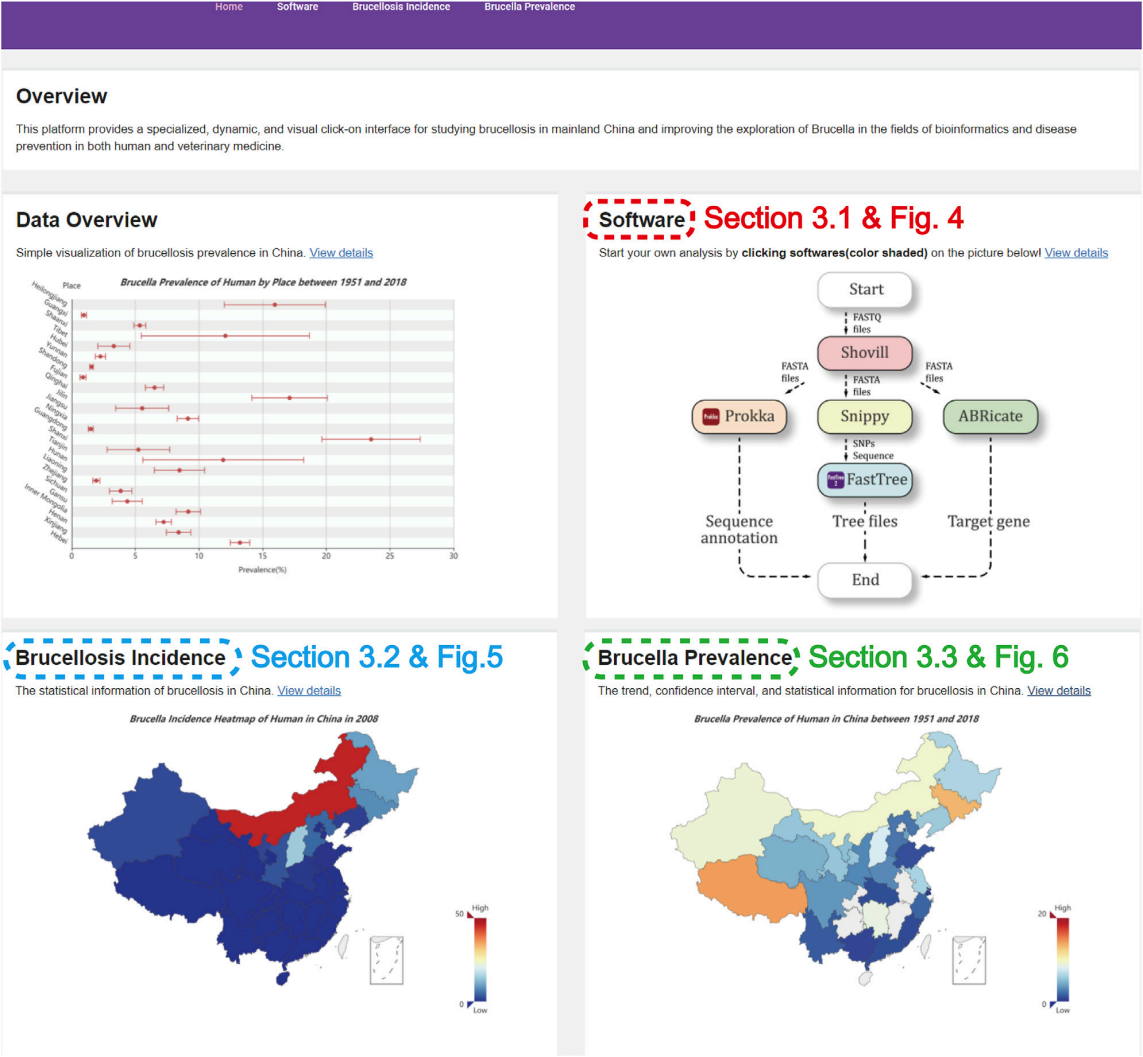
The homepage of the website.

1) The software package is based on a combination of multiple visualized genome sequence analytic software programs for *Brucella*, allowing the splicing of the genome with Shovill (version 1.1.0), the annotation of genomes by Prokka (version 1.14.5), the identification of single nucleotide polymorphisms by Snippy (version 4.6.0), the location the antimicrobial resistance genes by ABRicate (version 1.0.0), and the construction of an evolutionary tree with FastTree (version 2.1.9); this combination provides users with a specialized software package to compute and analyse *Brucella*-related data with a point-and-click interface (Figure 1).
2) There is a visual interface for the incidence (reported disease cases) and the prevalence (detected cases) of brucellosis in China, that presents the spatiotemporal distribution of brucellosis incidence and the aggregation of each region using a map based on the years and hosts and incorporates the dynamic evolution and chronological prevalence of brucellosis in humans and animals through previous data collected from the literature; this allows the analysis of an epidemic situation of human and animal brucellosis in dynamic time (Figure 1).

Figure 1 describes the homepage of the comprehensive information platform of *Brucella*, which consists of an “Overview” and three functional modules.

The “Overview” introduces our work regarding *Brucella*, which shows the prevalence distribution of human brucellosis and animal brucellosis in various provinces of China. The “Software” module has five software programs for *Brucella* genome analysis and is detailed in Section 3.1. The “Brucellosis Incidence” module presents the spatial distribution of brucellosis incidence in mainland China and the aggregation of each region in map form and is detailed in Section 3.2. The “*Brucella* Prevalence” module visualizes and presents the brucellosis prevalence in mainland China using previous data collection based on the literature and is detailed in Section 3.3.

Overall, this platform integrates professional analytic software to provide easy, efficient, and rapid data analysis operations for the *Brucella* genome while also visualizing the incidence and prevalence of brucellosis in mainland China in an intuitive and interactive format. Moreover, this platform could provide high-performance computational extensions for future therapeutic research on brucellosis and may have advantages for brucellosis source tracking in the future.

## 2 Materials and methods

### 2.1 Website development

The Integrated Platform for *Brucella* was developed on the Linux server (CentOS 7.5.1804), which employs MySQL (version 8.0.20) as the database server. The back and front ends of the website are based on the Django framework (version 3.1.1) and bootstrap framework (version 4.4.1), respectively. We deployed the website (Figure 1) on http://www.combio-lezhang.online/brucella/index/to provide open access.1.2 Data collection and preprocessing.

The annual data on the incidence of human brucellosis are from the China Health Statistics Yearbook (Supplementary Table S1).

The annual data of the incidence of animal brucellosis are from the Qingdao Municipal Health Commission; incidence is calculated by the number of animals (stock) and the number of cases (Supplementary Table S2).

The *Brucella* prevalence data were collected from the College of Animal Sciences, Zhejiang University (Zhou et al., 2020), covering a total of 14,005 articles (Supplementary Table S3). These articles included 12,723 Chinese articles and 1,282 English articles, all of which were published before July 2018.

**FIGURE 2:**
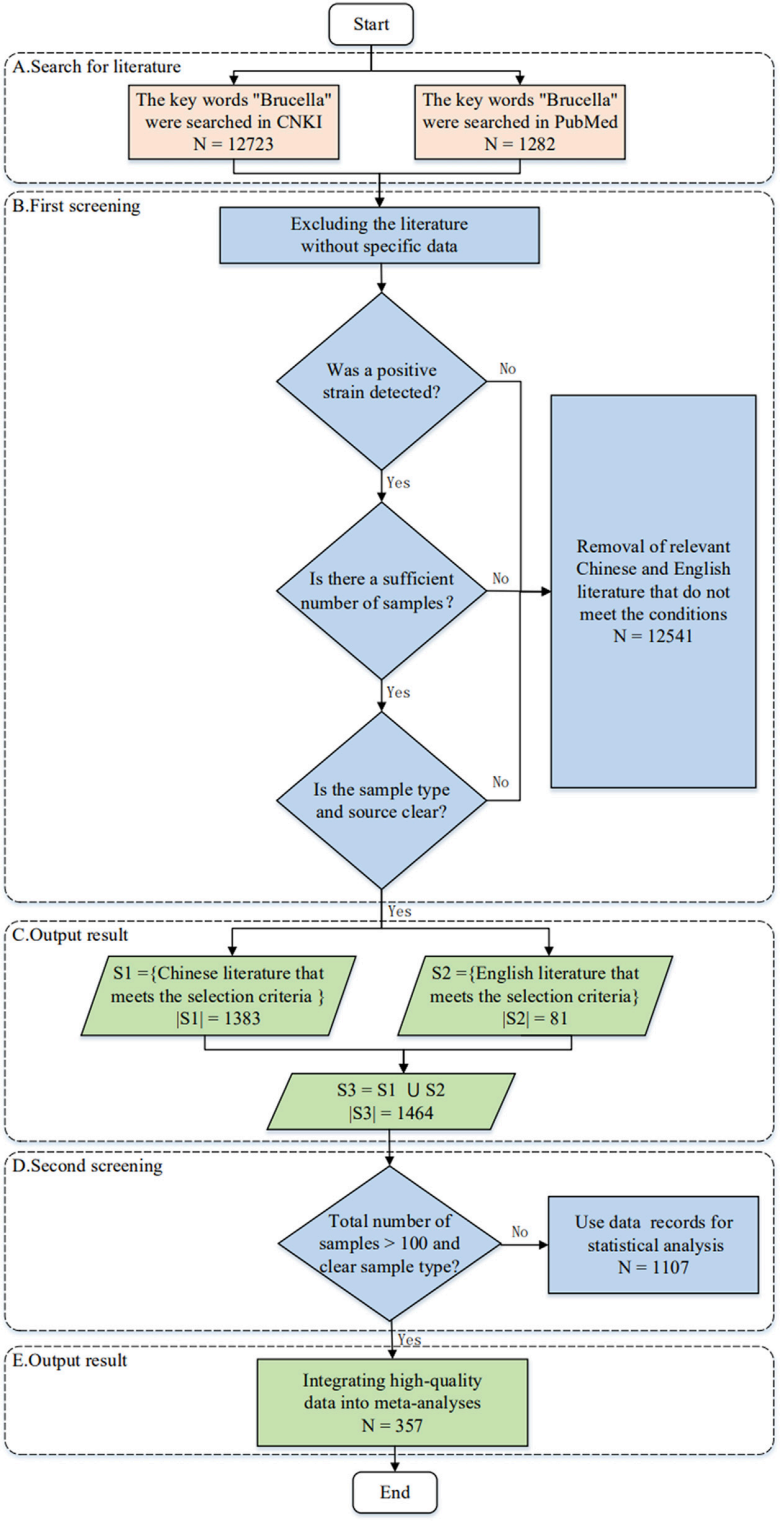
Flow chart for literature collection.

Figure 2 shows how to collect and organize *Brucella* prevalence information from 1,405 articles, which includes 357 high-quality articles and 688 data items related to this topic. Considering the significant discrepancies of different epidemiological studies in publication information, subjects, backgrounds and results (Messina et al., 2017; Xu et al., 2020), our study also establishes a knowledge graph for the literature sources of *Brucella* data so that it can be expanded, queried, and analyzed. Details of the knowledge graph and datasets can be accessed or downloaded from http://www.combio-lezhang.online/brucella/sample_page/.

### 2.2 Computing methods for *Brucella*-related data

**FIGURE 3:**
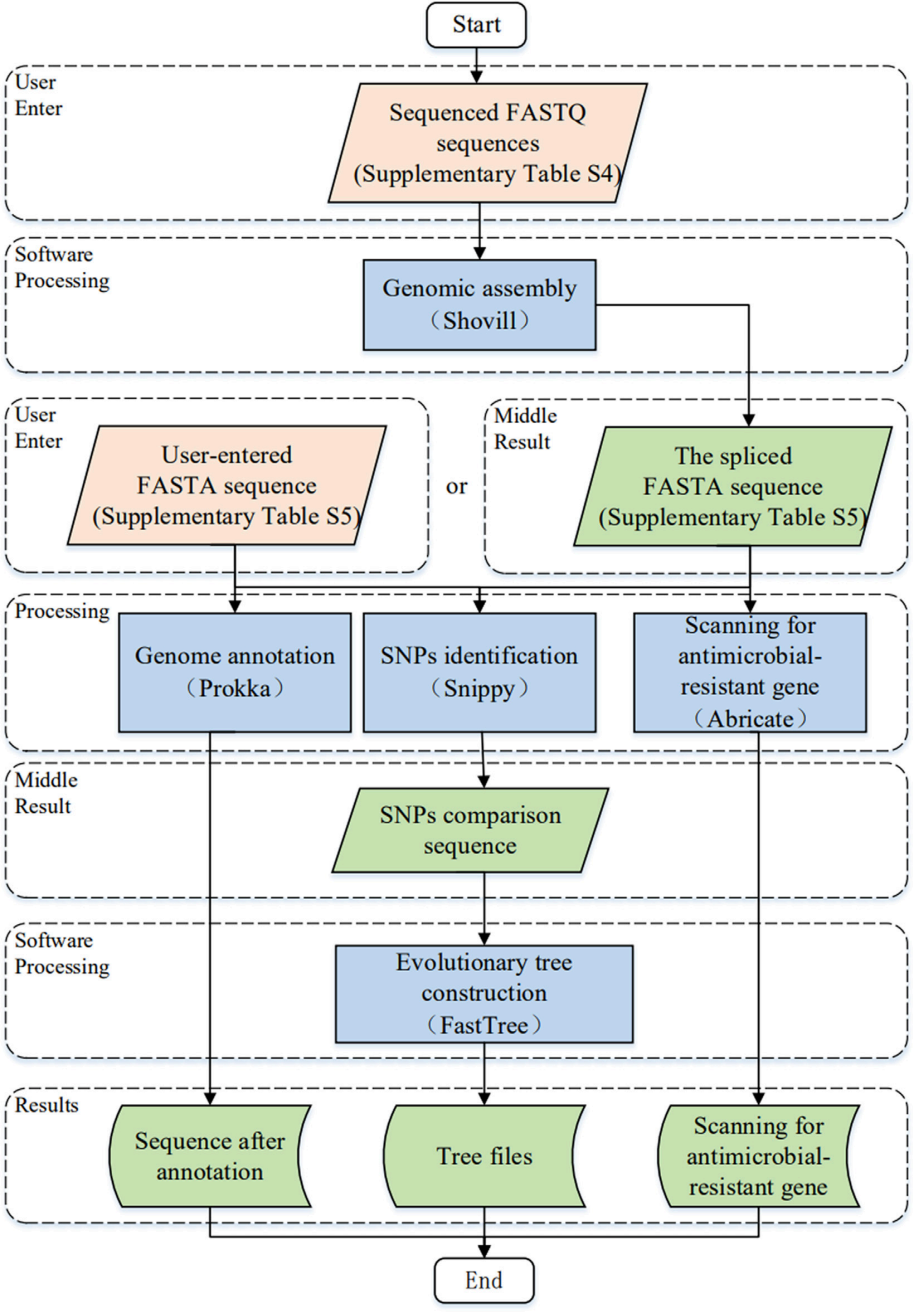
The workflow for software module development.

The study of the genome of *Brucella* can help us understand its interspecies and intraspecies evolutionary relationships and plays an important role in screening important genes and investigating their genetic mechanisms. The “Software” module integrates five genomic analysis software programs. Figure 3 illustrates the software workflow of the comprehensive information platform of *Brucella* as follows. First, users upload the sequenced FASTQ sequence file or FASTA sequence file onto the website and use the corresponding genome software step by step. The formats of the FASTQ and FASTA sequences are listed in Supplementary Tables S4, S5, respectively. The resulting file can be downloaded as a zip archive file.

With respect to the study of *Brucella* (Zhou et al., 2020), we chose five commonly used applications (Shovill, Prokka, Snippy, ABRicate and FastTree) for data processing. Figure 3 illustrates the key input parameters of the platform based on the developed comprehensive information platform of *Brucella*; the advanced features for each genome analysis software program are detailed by Shovill (Seemann, 2018), Prokka (Seemann, 2014), Snippy (Seemann, 2019), ABRicate (Seemann, 2017) and FastTree (Price et al., 2009).

1) Genomic assembly: Shovill (version 1.1.0) is a pipeline for the assembly of bacterial isolate genomes from Illumina paired-end reads, which is detailed in section 4.1.1.
2) Genome annotation: Prokka (version 1.14.5) is used to provide genome annotation services, which are detailed in section 4.1.2.
3) Identification of single nucleotide polymorphisms: Snippy (version 4.6.0) is used for SNP identification, which is described in detail in section 4.1.3.
4) Scanning for antimicrobial resistant genes: ABRicate (version 1.0.0) is used to scan the antimicrobial resistant genes, which is detailed in section 4.1.4.
5) Evolutionary tree construction: FastTree (version 2.1.9) is used to provide the phylogenetic tree construction service, which is detailed in section 4.1.5.

### 2.3 Computing methods for the incidence of *Brucella*

The workflow for brucellosis incidence development can be referred on Supplementary Figure S1. Here, we use local Moran’s I to carry out an autocorrelation analysis of brucellosis on local areas, which is a commonly used method in the field of public health (Zhang et al., 2013; Chen et al., 2017; Yin et al., 2018). After data input, Eq. 1 is used to calculate the local Moran’s I (Waller and Gotway, 2004) for each region of mainland China. Next, these computed local Moran’s I value are input into Eqs 2–4 for z-score computations. Finally, the software package PySAL is used to compute the statistical significance of the local autocorrelation coefficients.

Equation. 1 is used to compute local Moran’s I.

$$\begin{array}{c} I_{i}=\frac{Y_{i}}{\frac{1}{n}\sum {\left(y_{i}-\bar{y}\right)}^{2}}\times \overset{n}{\underset{j=1}{\sum }}w_{ij}Y_{j} \end{array}$$ (1)

Here, $\bar{y}$ is the mean of the attribute values of all provinces, cities, and regions in mainland China; ${Y_{\;i}=y}_{\;i}-\bar{y}$ is the difference between the incidence rate of region i and the mean incidence rate; n is the total number of all provinces, cities, and regions in mainland China; and $w_{ij}$ is the weight of region i and region j . If area i borders area j , $w_{ij}$ is 1; otherwise, it is 0.

The investigation of the local clustering of *Brucella* incidence in China can be then realized by the Z-test (Upton and Cook, 2008) of local Moran’s I in region i (Eq. 2)

$$\begin{array}{c} Z_{i}=\frac{I_{i}-E\left(I_{i}\right)}{\sqrt{V\left(I_{i}\right)}} \end{array}$$ (2)

Where $E\left(I_{i}\right)$ represents for the expectation of local Moran’s I in region i (Eq. 3), and $V\left(I_{i}\right)$ represents for the variance of local Moran’s I in region i (Eq. 4).

$$\begin{array}{c} E\left(I_{i}\right)=-\frac{1}{n-1} \end{array}$$ (3)

$$\begin{array}{c} V\left(I_{i}\right)=E\left(I_{i}^{2}\right)-E{\left(I_{i}\right)}^{2} \end{array}$$ (4)

Under the assumption that local Moran’s I follows the normal distribution, it can be considered statistically significant when $\left|Z_{i}\right|>1.96$ (where p−value<0.05 ). In this case, a positive local Moran’s I indicates the clustering of the brucellosis incidence area with similar (high or low) values, and thus it is part of a cluster; similarly, a negative local Moran’s I indicates the dispersion the brucellosis incidence area, and thus, it is an outlier (Feng et al., 2019). Figure S1 indicates the results computed by local Moran’s I value, which are classified into four aggregation areas (HH, LL, HL, LH, detailed in Table 1).

**TABLE 1:** Illustration of HH, LL, HL and LH.

Attribute	Description
HH	The region is comprised of high aggregation area of brucellosis incidence
LL	The region is comprised of low aggregation area of brucellosis incidence
HL	The region is in a high aggregation area, but surrounded by low aggregation area of brucellosis incidence
LH	The region is in a low aggregation area, but surrounded with high aggregation area of brucellosis incidence

### 2.4 Computing methods for the prevalence of *Brucella*

Investigating the prevalence of brucellosis can provide scientific data for the prevention of susceptible populations and serve as a reference to monitor epidemic prevention and the decontamination of breeding sites for brucellosis.

The workflow of the prevalence visualization module development for *Brucella* is described on Supplementary Figure S2. First, users can input the time interval and host. The input is then checked as to whether the collected prevalence of *Brucella* data is met (Supplementary Table S3). If it is met, Eq. 5 is used to compute the prevalence rate for each time interval and region. Simultaneously, Eqs 6, 7 and the software package Statsmodels (Seabold and Perktold, 2010) are used to compute the confidence interval of the prevalence rate for each time interval and region. The software package Echarts (version 4.8.0) (Bond and Goguen, 2002) is then used for prevalence visualization.

Equation 5 is used to compute the prevalence (Beaglehole et al., 1993), where P is the prevalence of a certain time interval or a certain region, ∑m is the total number of positive samples in a certain time interval or a certain region, and ∑n is the total number of samples in a certain time interval or a certain region.

$$\begin{array}{c} P=\frac{\sum m}{\sum n} \end{array}$$ (5)

Equation. 6 is used to compute the prevalence for each test record *i*, where $P_{i}$ is the prevalence (Beaglehole et al., 1993) rate for record *i*, $m_{i}$ is the number of positive tests for record *i*, and $n_{i}$ is the total number of samples for record *i*. Eq. 7 is used to compute the corresponding standard error, where $S_{i}$ is the standard error for each record. Finally, Supplementary Figure S3 shows the pseudocode to compute the 95% confidence interval for the prevalence rate.

$$\begin{array}{c} P_{i}=\frac{m_{i}}{n_{i}} \end{array}$$ (6)

$$\begin{array}{c} S_{i}=\sqrt{\frac{P_{i}\left(1-P_{i}\right)}{n_{i}}} \end{array}$$ (7)

## 3 Results

### 3.1 Software interface overview

In order to start a new *Brucella* analysis, users can click on the “software” link at the top of each page or the button of specific software on the flowchart (Figure 1).

**FIGURE 4:**
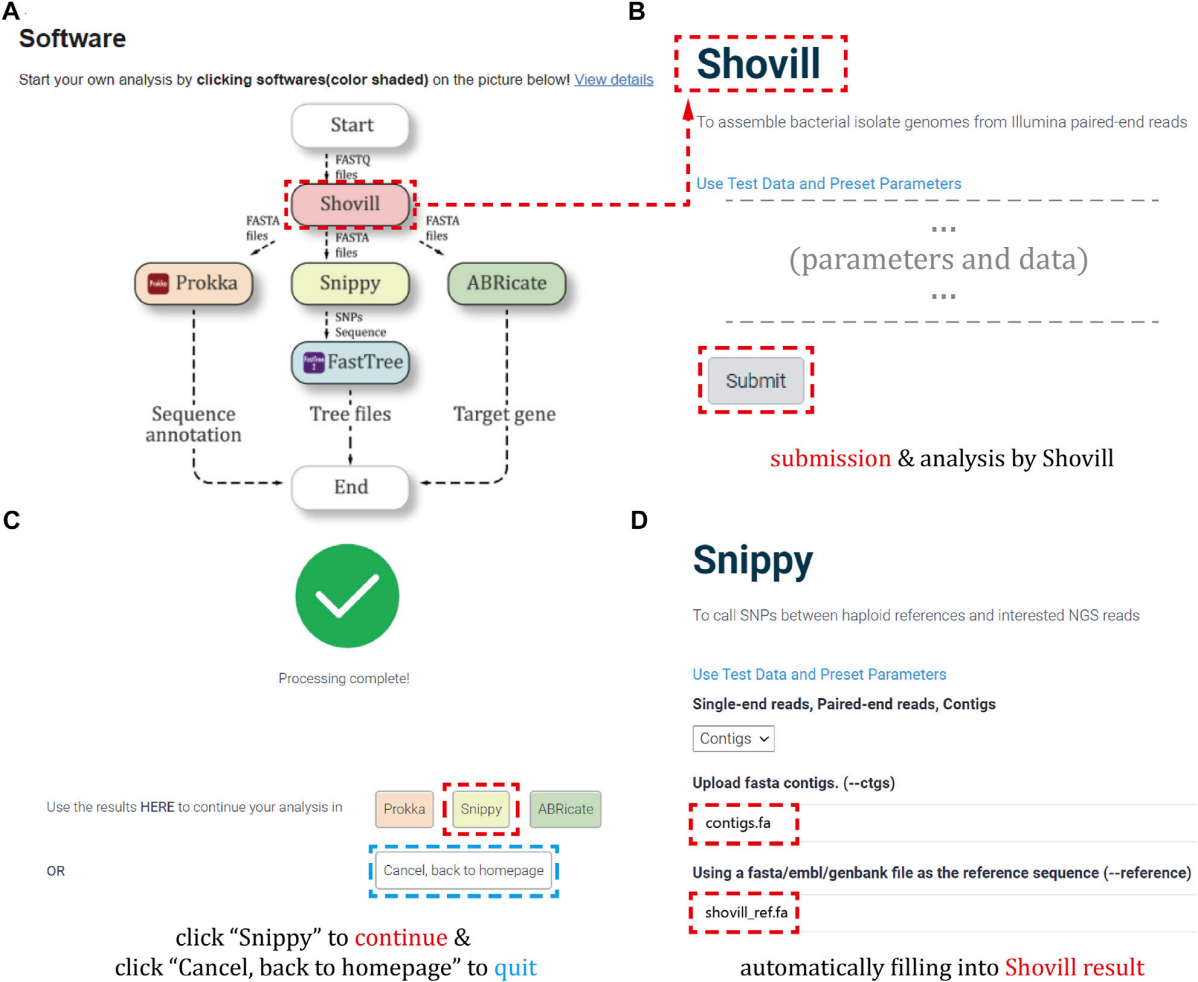
Analysis workflow. **(A)** The flowchart of the software analysis procedures on the homepage. **(B)** The example of starting a genomic analysis by data submission and parameter selection. **(C)** The interface of selecting corresponding software to continue the workflow when the analysis is completed. **(D)** The example of automatically filling results from previous step to the current software.

Here, Figure 4A describes the analysis procedure. Especially, Figures 4B–D show our developed “automatic data filling function”, which can avoid the inconvenience of repeatedly submitting result files during a single analysis.

Next, we will sequentially introduce the background and the requested parameters for each software integrated in the platform (Supplementary Figures S4–S8).

#### 3.1.1 Genomic assembly: Shovill

Shovill can help users assemble bacterial isolate genomes from Illumina paired-end reads for further comparison and analysis in the graphical tool of the phylogenetic tree (Seemann, 2018), which is described in Figure S4. Shovill uses SPAdes at its core, but alters the steps before and after the primary assembly step to obtain the similar results in less time.

Shovill shows 7 parameters. Among them, users must input “Forward reads (R1)” and “Reverse reads (R2)” entries. For example, users should upload the sequence files obtained by paired-end sequencing onto “Forward reads (R1)” and “Reverse reads (R2)”.

For another 5 parameters, including “Trim reads”, “Contig name format”, “Depth”, “Minimum contig length”, and “Minimum contig coverage”, if users click the sample file option “Use Test Data and Preset Parameters”, they can use the default parameters.

After users complete the parameter setting and click the “Submit” button, the system will generate 5 files in the specified output directory, which are shown in https://github.com/Rainbow-24/Brucella-supplementary. Each file has a common prefix, listed within Supplementary Table S6.

#### 3.1.2 Genome annotation: Prokka

Prokka can help users quickly annotate the genomes of bacteria, archaea and viruses for further analysis or viewing in the genome browser and produce standards-compliant output files (Seemann, 2014), which is described in Supplementary Figure S5.

Prokka shows 15 parameters. Among them, users must input “Contigs to annotate” and “Kingdom (--kingdom)” entries. For example, users should upload the FASTA genome sequence file onto the “Contigs to annotate” and select the species type for “Kingdom (--kingdom)”.

For another 13 parameters, including “Locus tag prefix (--locustag)”, “Locus tag counter increment (--increment)”, “GFF version (--gffver)”, “Force GenBank/ENA/DDJB compliance (--compliant)”, “Add ; features for each “CDS” feature (--addgenes)”, “Minimum contig size (--mincontiglen)”, “Sequencing centre ID (--centre)”, “Genus name (--genus)”, “Species name (--species)”, “Strain name (--strain)”, “Plasmid name or identifier (--plasmid)”, “Use genus-specific BLAST database (--usegenus)”, and “Optional FASTA file of trusted proteins to first annotate from (--proteins)”, users can select the default parameters by clicking the sample file option Use Test (--Data and Parters)”.

After users complete the parameter setting and click the “Submit” button, the system will generate 12 files in the specified output directory, which are shown in https://github.com/Rainbow-24/Brucella-supplementary. Each file has a common prefix, listed within Supplementary Table S7.

#### 3.1.3 Identification of single nucleotide polymorphisms: Snippy

Snippy can help users find SNPs between a reference genome and NGS sequence reads. Snippy will use as many CPUs as researcher can give it on a single computer (tested to 64 cores), and it is designed with speed in mind, and produces a consistent set of output files, including a core SNP alignment, in a single folder (and ultimately a phylogenomic tree) (Seemann, 2019), which is described in Supplementary Figure S6.

Snippy shows that users need to input 5 parameters. Among them, users need mandatory to input “Upload fasta contigs. (--ctgs)” and “Using a fasta/embl/genbank file as the reference sequence (--reference)” entries. For example, users should upload the FASTA genome sequence file onto the “Upload fasta contigs. (--ctgs)” and a reference sequence onto the “Using a file as the reference sequence (--reference)”.

For another 3 parameters, such as “Single-end reads, Paired-end reads, Contigs”, “Minimum mapping quality”, “Minimum coverage”, if users click the sample file option “Use Test Data and Preset Parameters”, they can use default parameters.

After users complete the parameter setting and click the “Submit” button, the system will generate 16 files in the specified output directory, which are in https://github.com/Rainbow-24/Brucella-supplementary. Each file has a common prefix, listed within Supplementary Table S8.

#### 3.1.4 Scanning for antimicrobial-resistant genes: ABRicate

ABRicate, which comes bundled with multiple databases (NCBI, CARD, ARG-ANNOT, Resfinder, MEGARES, EcOH, PlasmidFinder, Ecoli_VF and VFDB), can help users mass screen contigs for antimicrobial resistance or virulence genes (Seemann, 2017), as described in Supplementary Figure S7. Abricate was used in monitoring the spread of multidrug-resistant genes (Arnott et al., 2018) and searching for drug-resistant plasmid carriage in *E. coli (* *Zong et al., 2018* *).*

ABRicate shows 5 parameters. Among them, users must input “Input file (Fasta, GenBank or EMBL file)” and “Database to use - default is ‘resfinder'” entries. For example, users should upload the genome sequence file of antimicrobial resistant genes into the “Input file (Fasta, GenBank or EMBL file)” and select the database for “Database to use - default is “resfinder”.

For another 3 parameters, such as “Suppress header“, “Minimum DNA %identity (0–100)“, “Minimum DNA %coverage (0–100)“, if users click the sample file option “Use Test Data and Preset Parameters“, they can use default parameters.

After users complete the parameter setting and click the “Submit“ button, the system will generate a tap-separated in the specified output directory, which is in https://github.com/Rainbow-24/Brucella-supplementary. It contains multiple columns, listed within Supplementary Table S9.

#### 3.1.5 Evolutionary tree construction: FastTree

FastTree can help users infer approximate maximum-likelihood phylogenetic trees from the alignments of nucleotide or protein sequences to further estimate their reliability (Price et al., 2009), which is described in Supplementary Figure S8. For large alignments, FastTree is 100–1,000 times faster than previous methods, and is much more accurate than the distance-matrix methods that are traditionally used for large alignments.

FastTree shows 6 parameters. Among them, users must input “FASTA file“ and “Protein or nucleotide alignment“ entries. For example, users should upload a multiple sequence alignment file onto the “FASTA file” and select the sequence alignment type for “Protein or nucleotide alignment”.

For another 4 parameters, including “Aligned sequences file (FASTA or Phylip format)”, “Allow spaces and other restricted characters (but not ') in sequence and quote names in the output tree”, “Set starting tree”, and “Nucleotide evolution model”, if users click the sample file option “Use Test Data and Preset Parameters”, they can use the default parameters.

After users complete the parameter setting and click the “Submit” button, the system will generate a Tree file in Newick format in the specified output directory, which is shown in https://github.com/Rainbow-24/Brucella-supplementary.

### 3.2 Visualization of brucellosis incidence

**FIGURE 5:**
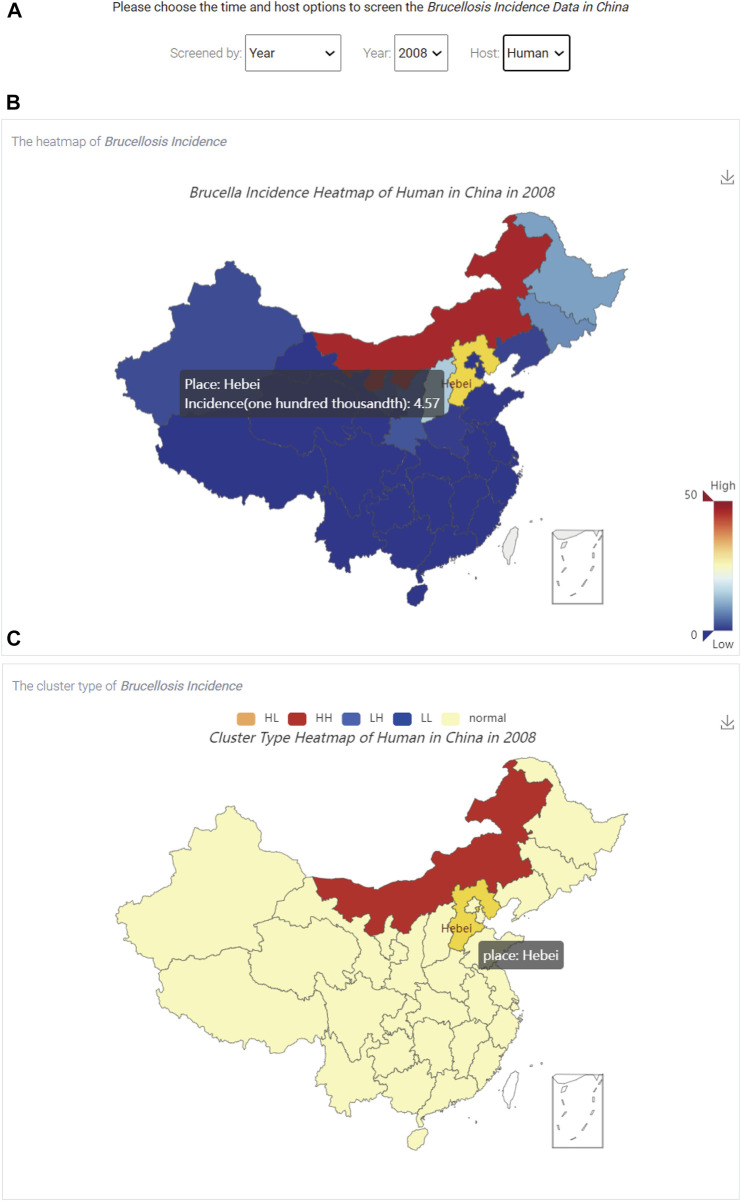
The visualization of brucellosis incidence in China. **(A)** The three option boxes; **(B)** The spatial distribution of brucellosis incidence; **(C)** The aggregation of brucellosis incidence.

The brucellosis incidence visualization module can show users the statistical information for brucellosis in China. After clicking the “Brucellosis Incidence” link on the homepage (Figure 1), the distribution and aggregation of brucellosis incidence in China can be queried, as shown in Figure 5.

Figure 5A shows that there are three options, including “Screened by”, “Year” and “Host” at the top of Figure 5, which represent the screening type (a specific year or a specific time interval), time and host, respectively. In addition, users can use the hyperlink in the upper right corner of Figure 5B to save the results in the specified output directory.

When users select the time and host, Figure 5B shows the spatial distribution of brucellosis incidence, and the colour of the box in the lower right corner represents the incidence rate from low to high. By hovering the mouse over different regions on the map of China, users can dynamically view the name of the region and brucellosis incidence data. Figure 5C shows the aggregation of brucellosis incidence. The five coloured boxes on the top represent the clustering or dispersion of brucellosis incidence, which are described in Table 1.

### 3.3 Visualization of *Brucella* prevalence

**FIGURE 6:**
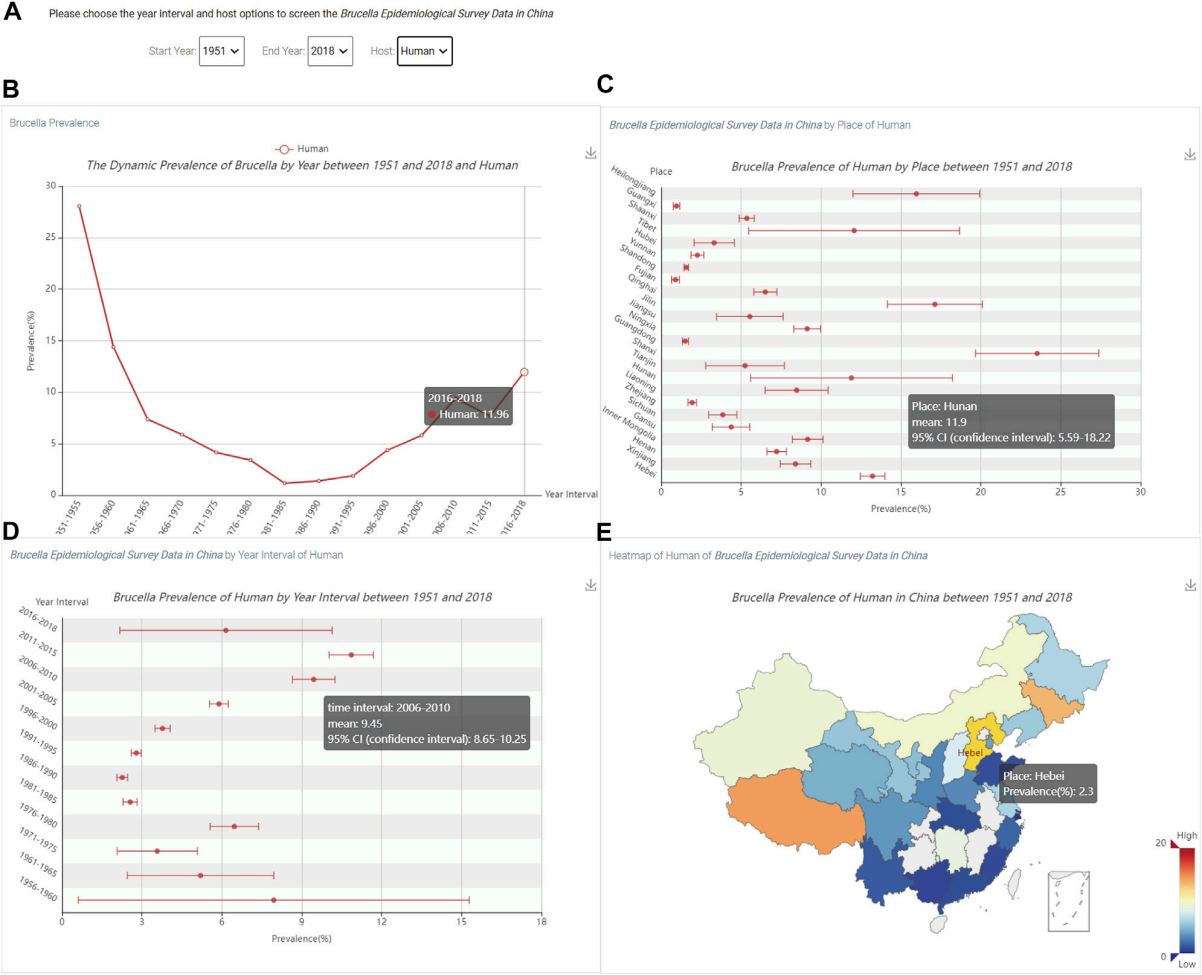
The visualization of the prevalence of *Brucella* in China. **(A)** The three option boxes; **(B)** The trend of human *Brucella* prevalence level; **(C)** The prevalence of *Brucella* by region; **(D)** The prevalence of *Brucella* for each time period; **(E)** The spatial distribution of the prevalence of *Brucella*.

The prevalence in the *Brucella* visualization module can show the trend, confidence interval, and statistical information for brucellosis in China. After clicking the “*Brucella* Prevalence” link on the homepage (Figure 1), the visualization of *Brucella* prevalence in China can be queried, as shown in Figure 6.

Figure 6A shows three options: “Start Year”, “End Year” and “Host”. In addition, users can use the hyperlink in the upper right corner of Figures 6B–E to save the results in the specified output directory.

When users select the time and host, Figure 6B shows the trend of human *Brucella* prevalence level; the *X*-axis is the time period and *Y*-axis is the percent prevalence. By hovering the mouse over different time periods, users can dynamically view the *Brucella* prevalence by time period. Figure 6C shows the *Brucella* prevalence for each region; the *X*-axis is the percent prevalence, and the *Y*-axis is the name of each province in China. By hovering the mouse over different regions, users can dynamically view the province name, mean prevalence and 95% confidence interval. Figure 6D shows the *Brucella* prevalence for each time period; the *X*-axis is the percent prevalence, and the *Y*-axis is the time period. By hovering the mouse over different time periods, users can dynamically view the time period, mean prevalence and 95% confidence interval. Figure 6E shows the spatial distribution of *Brucella* prevalence, and the colour of the box in the lower right corner represents the prevalence from low to high. By hovering the mouse over different regions on the map of China, users can dynamically view the prevalence of *Brucella* for individual regions.

## 4 Discussion and conclusion

To the best of our knowledge, there is no previous visualization website for brucellosis (or other infectious agents) in China or elsewhere in the world that provides a framework to query the trend and predicted direction of the incidence of infectious diseases.

Therefore, we developed this platform to integrate five commonly used genomic applications for convenient *Brucella*-related genomic analysis and to dynamically visualize the incidence (reported patients) and prevalence (detected cases) of brucellosis in mainland China.

Especially, our study establishes a knowledge graph for the literature sources of *Brucella* data so that it can be expanded, queried and analyzed. When similar “epidemiological comprehensive platforms” are established in the distant future, we can use knowledge graph to share its information.

Importantly, the current incidence dataset (the reported cases) and the prevalence dataset, (the detected positive cases) have generally good agreement for the different geographic regions (Supplementary Figures S9, S10).

Moreover, the newly developed, national-scale database for *Brucella* could offer an essential toolkit for primary genomic analysis and, more importantly, establish a framework to understand the burden of brucellosis regarding humans vs animals in a dynamic way, over a long period of time (1953–2018), for 31 different geographic locations.

However, the current platform can only provide a reference for the genomic analysis process and cannot be fully embedded into all aspects of the bioinformatics analysis process; data on the incidence and prevalence of brucellosis are relatively fixed and do not allow for real-time updates of relevant data.

For these reasons, we will apply more sophisticated bioinformatics applications (Zhang et al., 2017a; Zhang et al., 2017b; Gao et al., 2017; Li et al., 2017; Xia et al., 2017; Zhang and Zhang, 2017; Zhang et al., 2018; Zhang et al., 2019b) to brucellosis research in the future to improve the scalability of the platform’s algorithms and the projection of dynamic data.

## Data Availability

The datasets presented in this study can be found in online repositories. The names of the repository/repositories and accession number(s) can be found in the article/Supplementary Material.
