# Supplementary material for: An integrated platform for Brucella with knowledge graph technology: From genomic analysis to epidemiological projection
Source: Front Genet. 2022 Sep 14;13:981633. doi: 10.3389/fgene.2022.981633 (PMC9516312; doi:10.3389/fgene.2022.981633)
Supplement: Supplementary file 1 [file DataSheet1.docx]

Supplementary Material

# Supplementary Figures


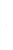

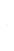

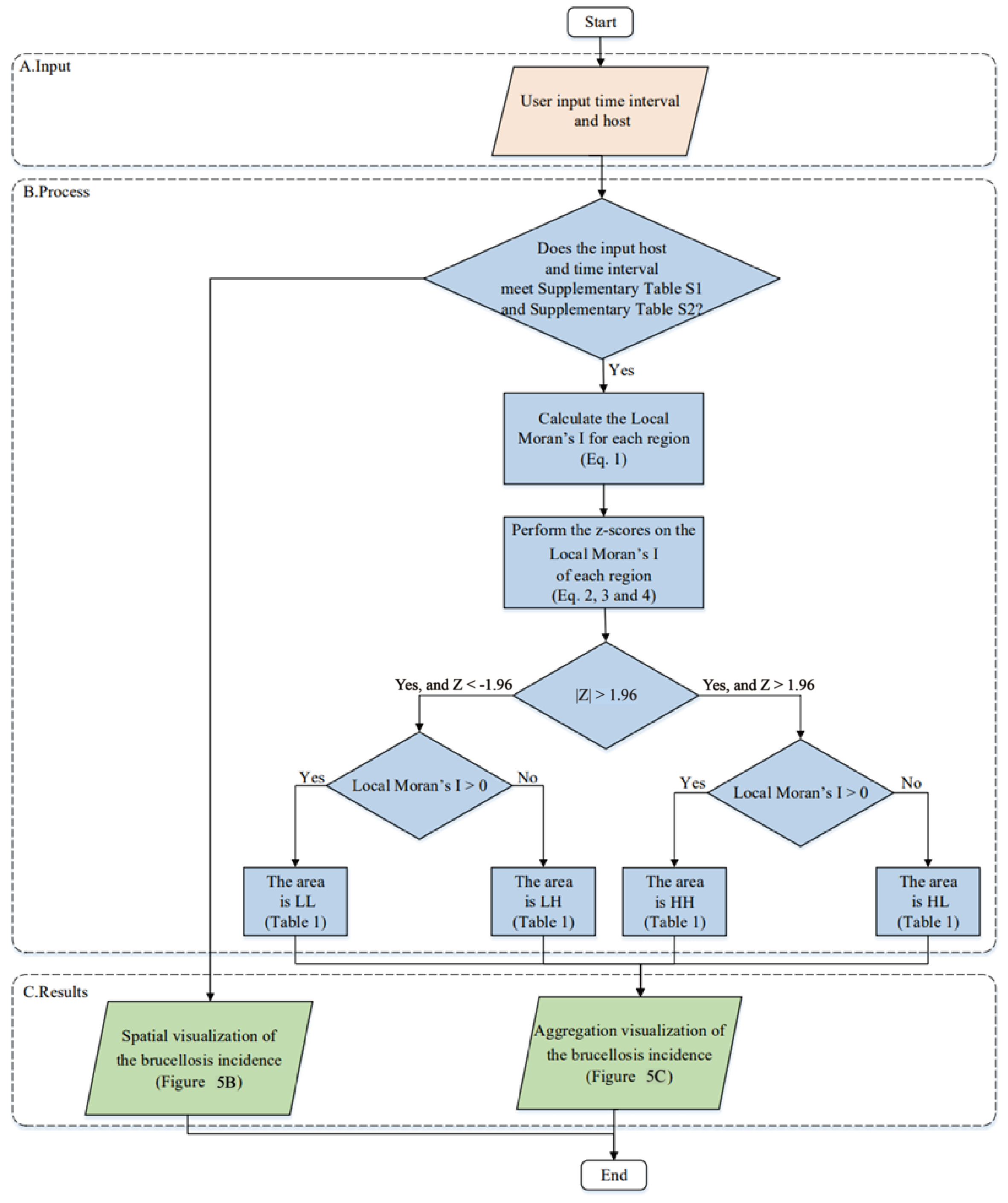


**Supplementary Figure S1.** The workflow for brucellosis incidence development.

| **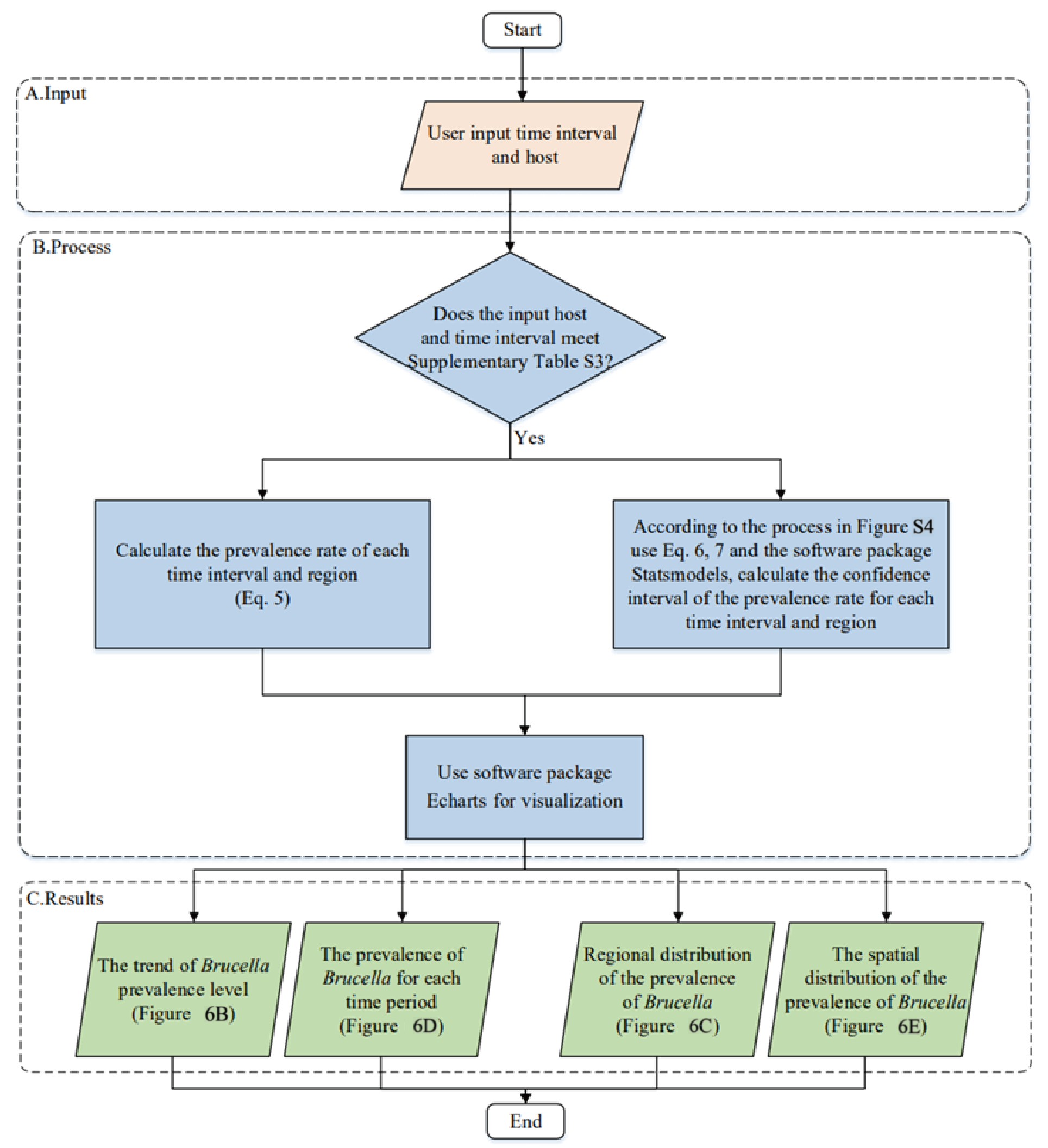**  **Supplementary Figure S2.** The workflow of the prevalence visualization module development for *Brucella.* |
| --- |


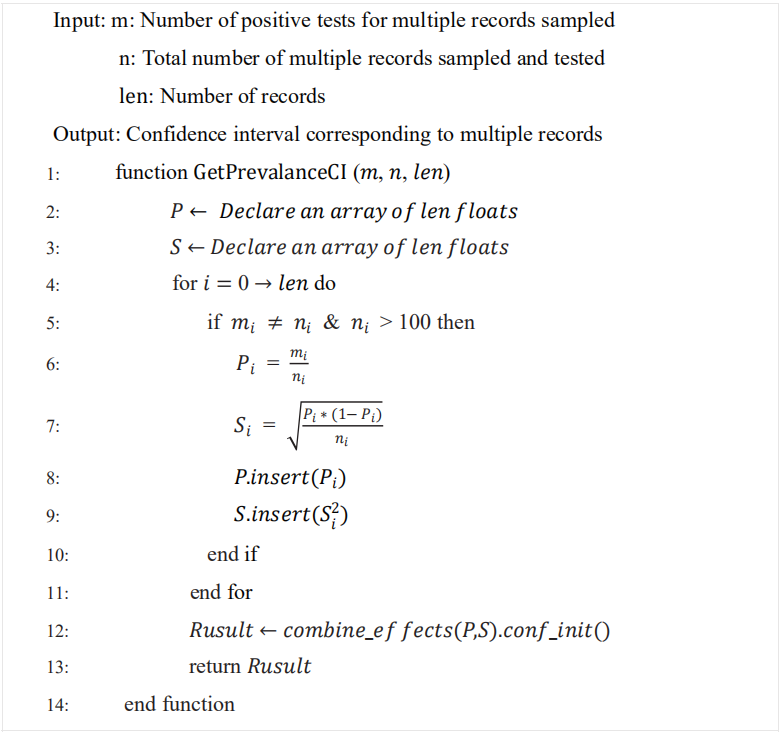


**Supplementary Figure S3.** The pseudo code of 95% confidence interval calculation process for prevalence. To make a comprehensive comparison between the brucellosis incidence and the prevalence of *Brucella*. We splitted the dataset from animal and human, and made a correlation study via geographical locations. We projected the data via geographical regions defined by China, and we observed a general higher prevalence of *Brucella* than the reported brucellosis incidence in China (**Supplemental Figure S9 and S10**). Due the heterogeneous nature for data origin and the aim of investigations, we observed a relative degree of inconsistence, indicating further cross-platform investigations by integrating data from human and animal to address the shared public health concern. Nevertheless, the data showed an overall consistence between the animals and humans among various of locations.

**
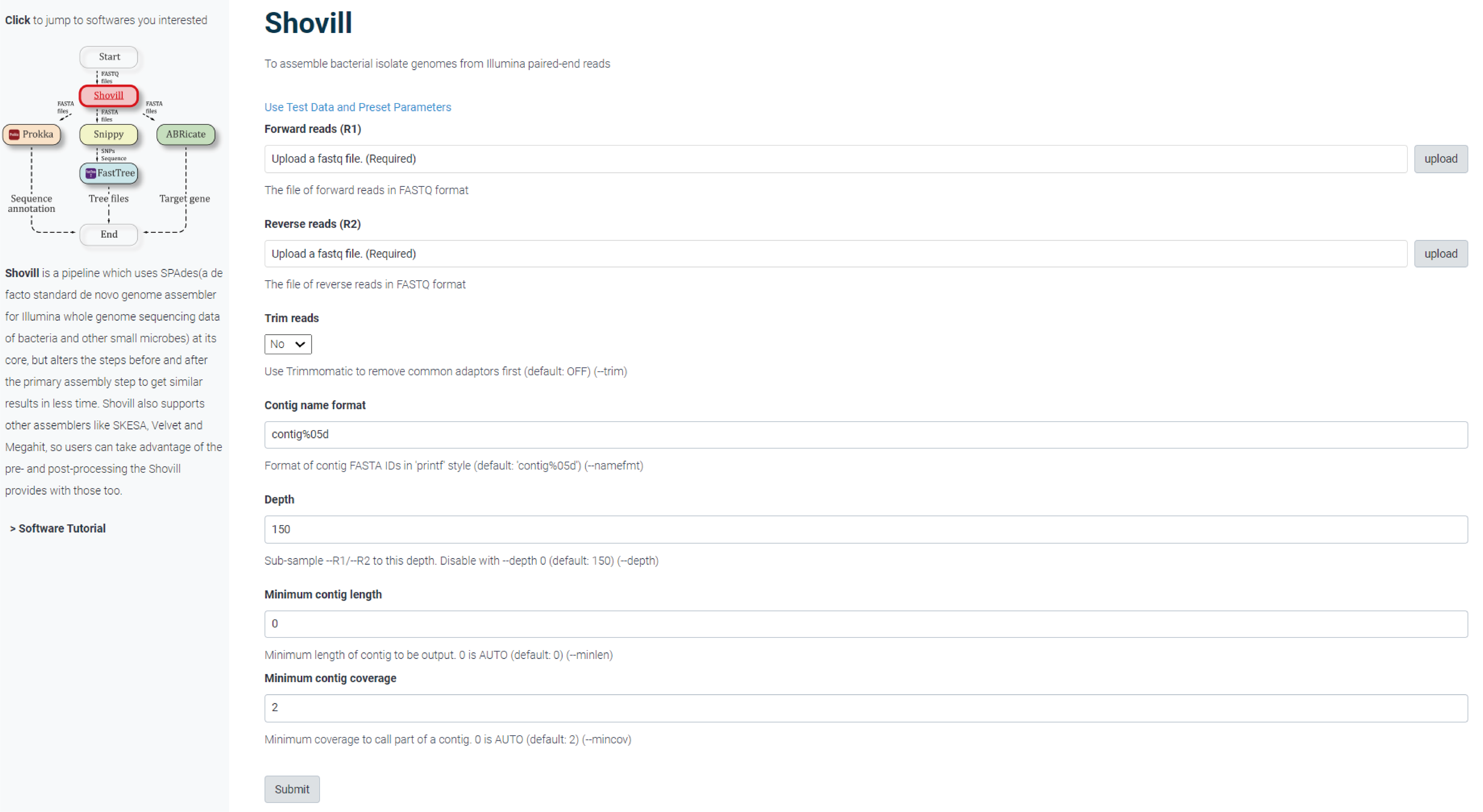
**

**Supplementary Figure S4.** Genomic assembly software “Shovill”





**Supplementary Figure S5.** Genome annotation software “Prokka”


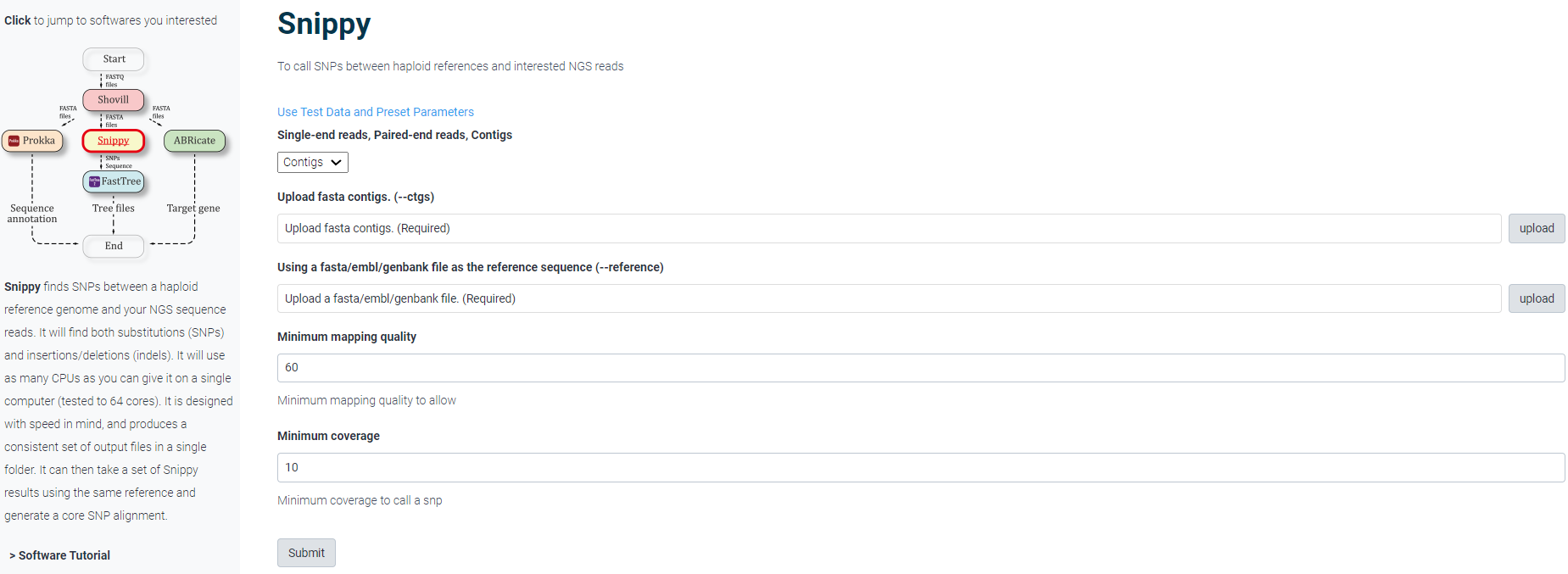


**Supplementary Figure S6.** SNPs identification software “Snippy”


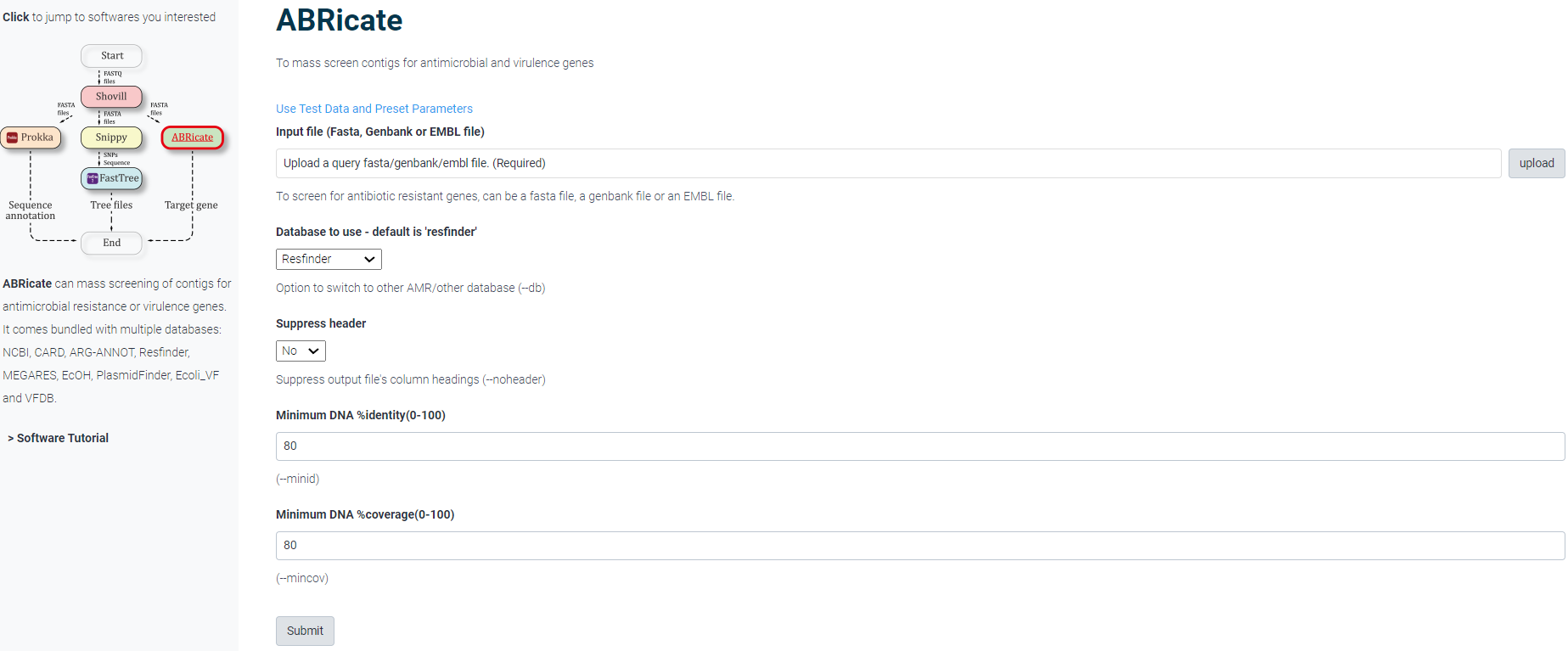


**Supplementary Figure S7.** Scanning for antimicrobial-resistant gene software “ABRicate”


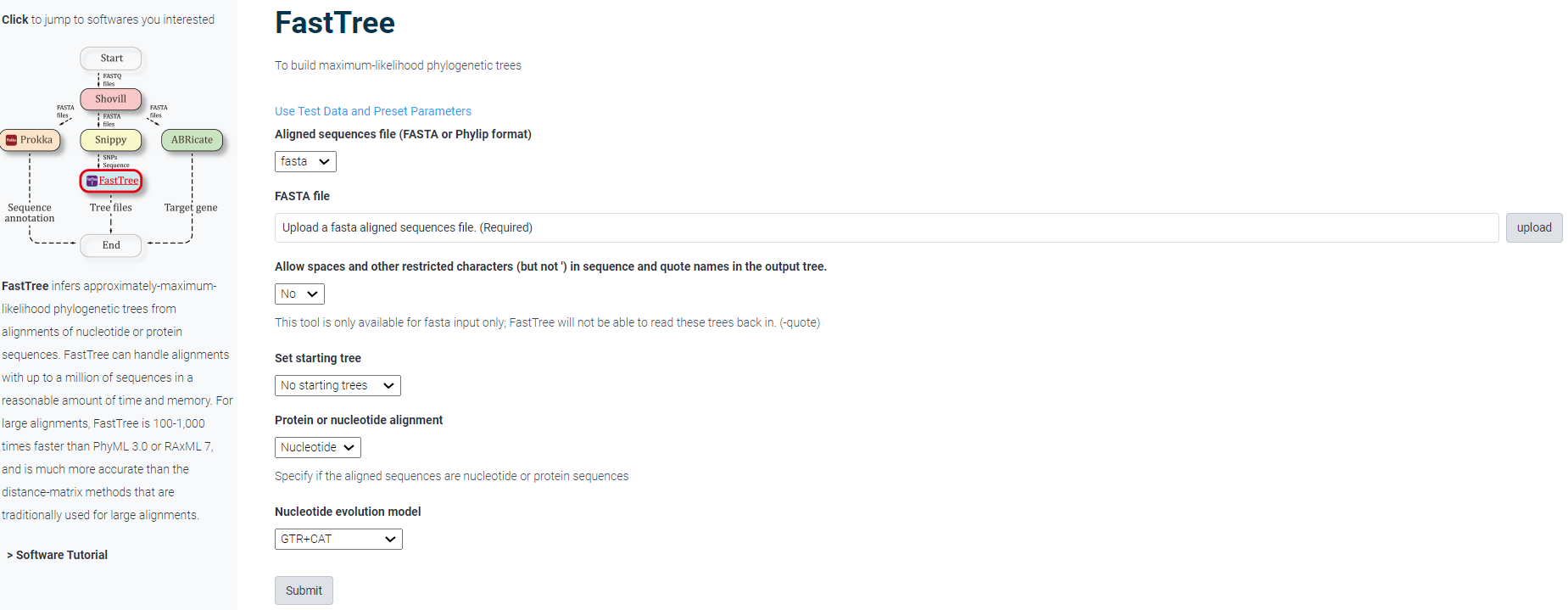


**Supplementary Figure S8.** Evolutionary Phylogenetic tree construction software “FastTree”


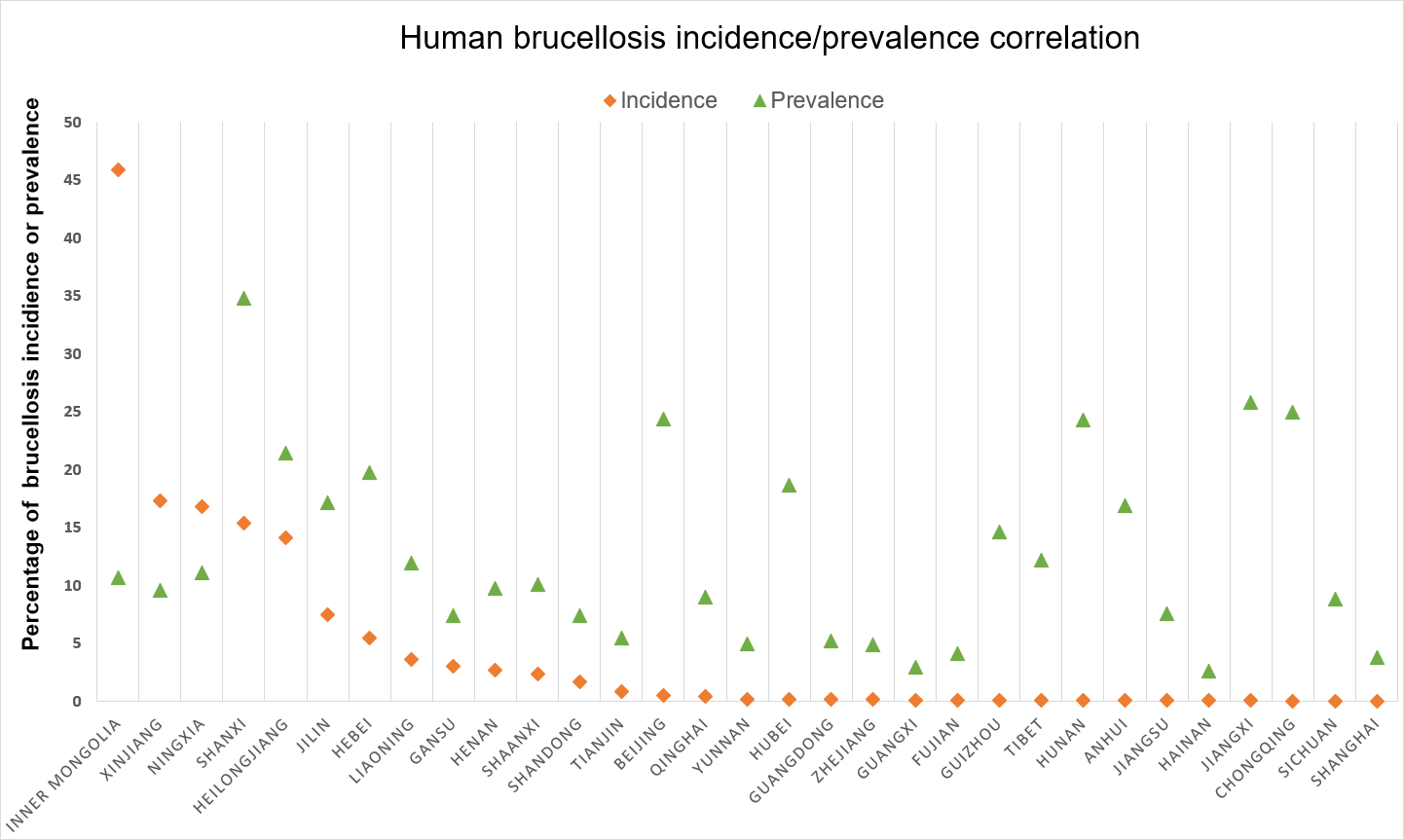


**Supplementary** **Figure S9.** A comparison between human brucellosis (orange) and the prevalence of Brucella (green) in humans.


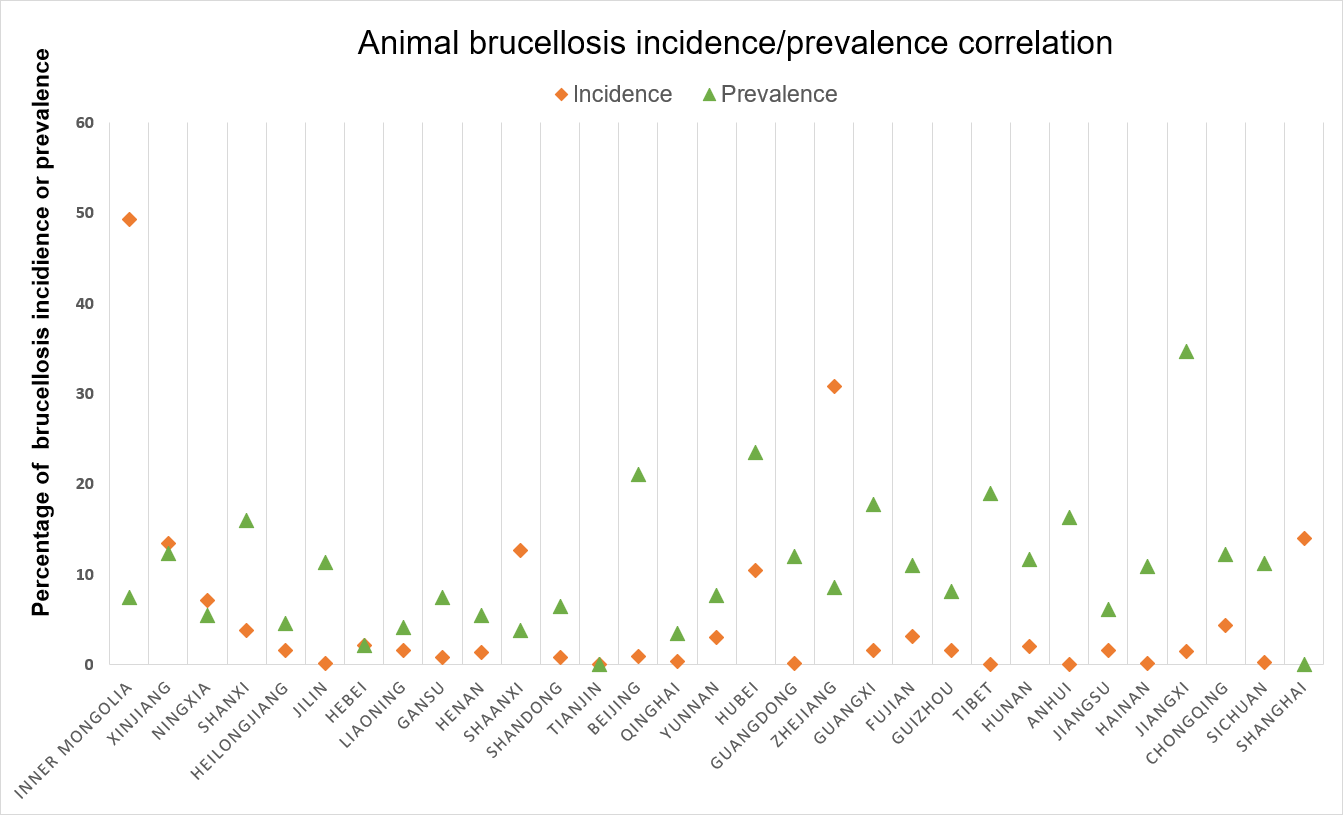
 **Supplementary** **Figure S10.** A comparison between animal brucellosis (orange) and the prevalence of *Brucella* (green) in humans.

# Supplementary Tables

**Supplementary Table S1.** The annual data of the incidence of human brucellosis in China.

**Supplementary Table S2.** The annual data of the incidence of animal brucellosis in China.

**Supplementary Table S3.** The data of *Brucella* prevalence in China.

**Supplementary Tables S1, S2 and S3** can be downloaded via <https://github.com/Rainbow-24/Brucella-supplementary>.

**Supplementary Table S4.** Example of FASTQ file.

| @SRR000001.2 EM7LVYS02GCAPL length=99 |
| --- |
| ACAGACTCAACCTGCATAATAAATAACATTGAAACTTAGTTTCCTTCTTGGGCTTTCGGTGAGAAAACATAAGTTAAAACTGAGCGGGCTGGCAAGGCN +SRR000001.2 EM7LVYS02GCAPL length=99 <=::=<8=D=C<<<<<<A=;CA1<=7<;A<;CA1 <@:<9>;&> 7;4<>7CA0<C@0:<5<;:<CA7+:7<@9<B=CA7+<<99<:B?.<;:2<A<A;:! |

**Supplementary Table S5.** Example of FASTA file.

| >CP000872.1 Brucella canis ATCC 23365 chromosome I, complete sequence |
| --- |
| TTTTCCACACTTATCCACAGGGCGCGGGCGGGACTCGGTTGCCCCTCTGAGTCAAGCATATTTTTTAAAATTATTTTTCCGGCAGAACATATCAGGATCGCCTAAAAATTGTAGCTGCGAAACACCTTGAATCCTAAGGTGTTATTTCGCTGGCATCCGATTCCCCAACAATGGTCCGTCTTCATGCAAGCCCTTTGCAGGAAAAAAATGAACGATCTGAGGCTTGCTCTTTGTGACGAGAGGTTGATTGCTTACCATTCATTAACTGGAATGCCGTTTAGGGCATCTCCGCGTCATTTGAGTGGTTGTGT |

**Supplementary Table S6.** Description of “Shovill” output files.

| Suffix | Description of file contents |
| --- | --- |
| shovill.log | Full log file for bug reporting |
| velvet.fasta | Raw assembly (velvet) |
| shovill.corrections | List of post-assembly corrections |
| contigs.LastGraph  contigs.fa | Assembly graph (velvet)  The final assembly you should use |

**Supplementary Table S7.** Description of “Prokka” output files.

| Suffix | Description of file contents |
| --- | --- |
| .gff | This is the master annotation in GFF3 format, containing both sequences and annotations. It can be viewed directly in Artemis or IGV. |
| .gbk | This is a standard Genbank file derived from the master .gff. If the input to Prokka was a multi-FASTA, then this will be a multi-Genbank, with one record for each sequence. |
| .fna | Nucleotide FASTA file of the input contig sequences. |
| .faa | Protein FASTA file of the translated CDS sequences. |
| .ffn | Nucleotide FASTA file of all the prediction transcripts (CDS, rRNA, tRNA, tmRNA, misc_RNA) |
| .sqn | An ASN1 format "Sequin" file for submission to Genbank. It needs to be edited to set the correct taxonomy, authors, related publication etc. |
| .fsa | Nucleotide FASTA file of the input contig sequences, used by "tbl2asn" to create the .sqn file. It is mostly the same as the .fna file, but with extra Sequin tags in the sequence description lines. |
| .tbl | Feature Table file, used by "tbl2asn" to create the .sqn file. |
| . err | Unacceptable annotations - the NCBI discrepancy report. |
| .log | Contains all the output that Prokka produced during its run. This is a record of what settings you used, even if the --quiet option was enabled. |
| .txt | Statistics relating to the annotated features found. |
| .tsv | Tab-separated file of all features: locus_tag, ftype, len_bp, gene, EC_number, COG,product. |

**Supplementary Table S8.** Description of “Snippy” output files.

| Suffix | Description of file contents |
| --- | --- |
| .tab | A simple tab-separated summary of all the variants. |
| .csv | A comma-separated version of the .tab file. |
| .html | A HTML version of the .tab file. |
| .vcf | The final annotated variants in VCF format. |
| .bed | The variants in BED format. |
| .gff | The variants in GFF3 format. |
| .bam | The alignments in BAM format. Includes unmapped, multimapping reads. Excludes duplicates. |
| .bam.bai | Index for the .bam file. |
| .log | A log file with the commands run and their outputs. |
| .aligned.fa | A version of the reference but with -at position with depth=0 and N for 0 < depth < --mincov (does not have variants). |
| .consensus.fa | A version of the reference genome with all variants instantiated. |
| .consensus.subs.fa | A version of the reference genome with only substitution variants instantiated. |
| .raw.vcf | The unfiltered variant calls from Freebayes. |
| .filt.vcf | The filtered variant calls from Freebayes. |
| .vcf.gz | Compressed .vcf file via BGZIP. |
| .vcf.gz.csi | Index for the .vcf.gz via bcftools index. |

**Supplementary Table S9.** Description of “ABRicate” output files.

| Column | Description of file contents |
| --- | --- |
| FILE | The filename this hit came from. |
| SEQUENCE | The sequence in the filename. |
| START | Start coordinate in the sequence. |
| END | End coordinate. |
| STRAND | Strand + or –. |
| GENE | AMR gene name |
| COVERAGE | What proportion of the gene is in our sequence. |
| COVERAGE_MAP | A visual represenation of the hit.$=$= aligned, .=unaligned, /=has_gaps. |
| GAPS | Openings / gaps in subject and query - possible psuedogene? |
| %COVERAGE | Proportion of gene covered. |
| %IDENTITY | Proportion of exact nucleotide matches. |
| DATABASE | The database this sequence comes from. |
| ACCESSION | The genomic source of the sequence. |
| PRODUCT | Gene product (if available). |
| RESISTANCE | putative antibiotic resistance phenotype, ;-separated |
